# Effect of Propolis on Dentin Regeneration and the Potential Role of Dental Pulp Stem Cell in Guinea Pigs

**Published:** 2011-12-22

**Authors:** Zohreh Ahangari, Mandana Naseri, Maryam Jalili, Yasaman Mansouri, Fatemeh Mashhadiabbas, Anahita Torkaman

**Affiliations:** 1. Department of Endodontic, Dental Research Center of Shahid Beheshti University of Medical Science, Tehran, Iran; 2. Private Practice, Tehran, Iran; 3. Department of Pathology, Dental Research Center of Shahid Beheshti University of Medical Science, Tehran, Iran; 4. Department of Physiology and Pharmacology, Pasteur Institute Tehran, Iran

**Keywords:** Dental Pulp Stem Cell, Dentin, Guinea Pigs, Propolis, Regeneration

## Abstract

**Objective:**

Evaluation of the effect of Propolis as a bioactive material on quality of dentin and presence of dental pulp stem cells.

**Materials and Methods:**

For conducting this experimental split-mouth study,a total of 48 maxillary and mandibular incisors of male guinea pigs were randomly divided into an experimental Propolis group and a control calcium hydroxide group. Cutting the crowns and using Propolis or calcium hydroxide to cap the pulp, all of the cavities were sealed. Sections of the teeth were obtained after sacrificing 4 guinea pigs from each group on the 10^th^, 15^th^ and 30^th^ day. After they had been stained by hematoxylin and eosin (H&E), specimens underwent a histological evaluation under a light microscope for identification of the presence of odontoblast-like cells, pulp vitality, congestion, inflammation of the pulp and the presence of remnants of the material used. The immunohistochemistry (IHC) method using CD_29_ and CD_146_ was performed to evaluate the presence of stem cells and the results were statistically evaluated by Kruskal-Wallis, Chi Square and Fisher tests.

**Results:**

In H&E stained specimens, there was no difference between the two groups in the presence of odontoblast-like cells, pulp vitality, congestion, inflammation of the pulp and the presence of remnants of used material(p>0.05). There was a significant difference between the quality of regenerative dentin on the 15^th^ and 30^th^ days (p<0.05): all of the Propolis cases presented tubular dentin while 14% of the calcium hydroxide cases produced porous dentin. There was no significant difference between Propolis and calcium hydroxide in stimulation of dental pulp stem cells (DPSCs).

**Conclusion:**

This study which is the first one that documented the stimulation of stem cells by Propolis, provides evidence that this material has advantages over calcium hydroxide as a capping agent in vital pulp therapy. In addition to producing no pulpal inflammation, infection or necrosis this material induces the production of high quality tubular dentin.

## introduction

Dental pulp is sometimes exposed during clinical procedures, such as cavity preparation or caries removal. In this situation, the pulp is involved in a process called reparative dentinogenesis, where some of the cells deposit a new matrix as a barrier in the injured site. It has been shown that adult dental pulp contains precursor cells capable of forming odontoblast-like cells in response to appropriate signals and materials (1). So, in certain cases, using direct pulp capping to save pulpal health and function is recommended. Since the selection of the capping material is a critical factor to produce the best treatment outcome, many studies of capping materials are carried out by researchers. The ideal properties of pulp capping agents are infection control, ease of handling, prevention of micro leakage and promotion of hard tissue formation (2). During the reparative process in exposed pulp primary odontoblasts that were lost are replaced with newly differentiated odontoblast-like cells. This process is known to follow the sequential steps of proliferation, migration and differentiation of progenitor or stem cells (3). It has been suggested that these newly formed cells were the pulp cells and undifferentiated mesenchymal cells. However Gronthos has reported the presence of a unique population of postnatal dental pulp stem cells (DPSC) with self-renewing, highly proliferative capacity and multipotential differentiation into odontoblast-like cells which formed the dentin matrix with some tubular features in vivo (4). Some other researchers have also identified a potential mesenchymal stem cell population derived from exfoliated deciduous human teeth, named as stem cells of human exfoliated deciduous (SHED) teeth, capable of extensive proliferation and multipotential differentiation to these cells (4, 5).

Various materials have been used in vital pulp procedures, especially direct pulp capping. Calcium hydroxide has been extensively and regularly used for direct pulp capping in modern clinical dentistry. As it is known to have a potential role in inducing hard tissue repair, this material has been applied to the exposed pulp and the hard tissue is expected to be regenerated over the pulp. The antimicrobial effect of calcium hydroxide relates directly to its high pH (12.5), which has destructive effect on cell membranes and protein structures. The action of calcium hydroxide is dependent on its dissociation and the release of hydroxyl ions (OH-), which diffuse into the surrounding tissues and result in the formation of a necrotic layer (6). The reparative dentin which is formed by calcium hydroxide is porous and is not a complete barrier. As the result, development of biocompatible materials that induce normal dentin-pulp complex is preferred (7).

Recently Propolis has been recognized as a useful material for human health and veterinary medicine. Made by the honeybee, it is a potent antimicrobial and anti-inflammatory agent. Honeybees collect the resin from cracks in the bark of trees and leaf buds (8). In general, Propolis is composed of 50% resin and vegetable balsam, 30% wax, 10% essential and aromatic oils, 5% pollen and 5% other various substances, including organic debris depending on the place and time of its collection (6, 9). The constituents of Propolis vary widely due to climate, season and location; so its chemical formula is not stable (10, 11). The most important pharmacologically active constituents in Propolis are flavonoids, which are well-known plant compounds that have antioxidant, antibacterial, antifungal, antiviral, and anti-inflammatory properties (6, 9-12). Studies of Propolis applications have increased because of its therapeutic and biological properties (9, 10). Current research involving Propolis in dentistry spans many fields, particularly in cariology, oral surgery, periodontics and endodontics due to its properties, especially its biocompatibility (9, 11- 13).

The main aim of this study was to evaluate the effect of Propolis on dentin regeneration and on the potential role of DPSCs.

## Materials and Methods

For conducting this experimental split-mouth study, dried Propolis collected in spring (from Azerbaijan) was subjected to exhaustive maceration, filtered using aqueous ethanol (96%) and concentrated using a rotary evaporator. The alcoholic extraction of Propolis was accomplished by repeating the above process three times. A total of 48 mandibular and maxillary incisors from 12 male guinea pigs (age 8-10 weeks, weight 200-250 g) were randomly divided into two groups; Propolis as the experimental and calcium hydroxide as control groups, each containing 24 teeth. The animals underwent general anesthesia with ketamine 60 mg/kg (ROTEXMEDICA, Germany) and xylazine 2%, 10 mg/kg (ALFASAN, Woerden, Holland) intra peritoneally. After the incisors had been cleaned and disinfected with 3% hydrogen peroxide followed by swabbing of the mouth with 0.2% chlorohexidinegluconate, the teeth were cut perpendicularly just above the level of the gingiva with a disk rotated by a low speed engine. Pulp exposure was performed using a number of 0.5 round dental burs and cavities of 1mm in diameter and 2mm in depth were prepared. During cavity preparation the tooth and cutting instruments were irrigated with sterile saline to prevent any heat damage generated by the process. The exposed pulp tissues of each group were directly capped with Propolis and calcium hydroxide, (Aria dent, Iran) respectively. The cavities were subsequently sealed with glass ionomer cement (Fuji II, GC, Japan). Four animals were sacrificed at 10, 15 and 30 days. Resected teeth and surrounding bone were fixed in 10% neutral formalin, demineralized in formic acid for 15 days, embedded in paraffin and sectioned serially in 4µm thickness parallel to the long axis of the tooth. The sections were stained using hematoxylin and eosin (H&E) and viewed by light microscopy. Histological evaluation was carried to determine vascular congestion, pulp vitality, presence of inflammatory cells, level of inflammation, presence of odontoblast-like cells, presence of dentinal bridge, quantity and quality of newly formed dentin and particles remaining from the capping material.

For the detection of DPSCs, the en-vision immunehistochemistry (IHC) method was conducted using the CD_29_ antibody, which is a specific marker for DPSC, and CD_146_, which is another DPSC and perivascular marker (14).

### Statistical analysis

Statistical analyses were conducted using the Kruskal-Wallis, Chi Square and Fisher tests. The analyses were performed using SPSS17.

### Ethical considerations

All procedures were conducted in accordance to the animal guidelines of the Pasteur Institute, Tehran, IRAN.

## Results

A total of 48 incisors from 12 guinea pigs were treated randomly with Propolis or calcium hydroxide as a pulp capping agent; with 24 teeth receiving each treatment. Vascular congestion was evident in both the experimental cases treated with Propolis and in the controls within different time periods. Throughout the experimental period all the cases treated with Propolis were observed to have vital pulp without any sign of necrosis, in contrast to the controls in which 75% had vital pulp during the same period. Chronic inflammation with dominance of lymphocyte and plasma cells was detectable in both groups at a level below 10%.

In all cases, odontoblast-like cells were present at every interval and there was no significant difference between the two groups (p>0.05). These cells were in an organized layer which could be seen either on the pulp chamber walls or under the formed reparative dentin.

True dentinal bridge did not form during the experimental period in either group. The dentin was formed in an irregular manner and in abundance.

**Fig 1:**
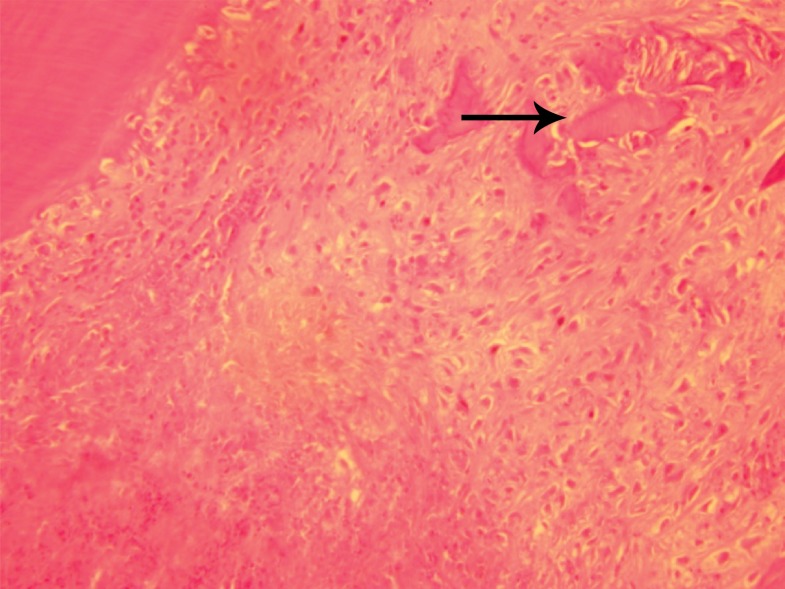
Porous dentin formation (arrow) in calcium hydroxide group (Magnification ×200)

**Fig 2:**
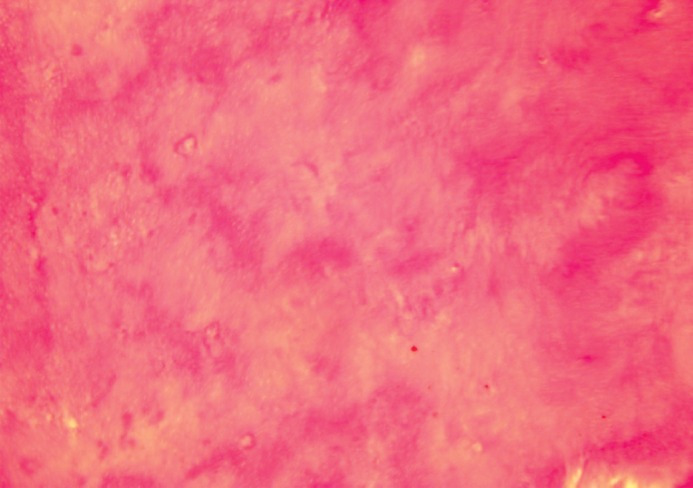
Tubular dentin formation in Propolis group (Magnification×200)

Although there was no significant difference between the quantities of dentin produced in both groups over the experimental period, this was not true of its quality. By the 10^th^ day there was no significant difference in the formation of tubular or porous dentin between the two groups. On day 15 those treated with Propolis had 70% tubular dentin while 90% of the control group had ?only? porous dentin (Fig 1). However, by day 30 all the cases (100%) with Propolis had tubular dentin (Fig 2) while only 86% of the control group had tubular dentin (p=0.005).

Within 15 days only 20% of the cases had remnants of Propolis while 40% of the controls had remnants of calcium hydroxide. However, by the 30^th^ day 17% of the experimental group still had remnants of Propolis, while there were no remnants of calcium hydroxide in the control group by this time.

The following results were obtained in the IHC evaluation, using CD_29_ and CD_146_ as markers for the detection of DPSCs. In both groups the presence of DPSCs either within the dentin or around the vessels was evident at the 10, 15 and 30-day examinations in both groups. Based on CD_29_ and CD_146_ markers there were more perivascular stem cells than stem cells in the dentin in both the experimental and the control group during the study.

Using information from both markers, fewer stem cells were detected in the calcium hydroxide group over time i.e. decreasing from 62.5% on the 10^th^ day to 20% in 30^th^ day and 54.5% to 20% for CD_146_ and CD_29_, respectively (Table 1). There was no specific pattern in the detection of stem cells in the Propolis group over time.

**Table 1:** The presence of DPSCs in Propolis and calcium hydroxide groups identified by CD_146_ and CD_29_ markers at different intervals over the experimental period


	CD_146_		CD_29_	
	DPSC presence		DPSC presence	
Time	Propolis	Ca(OH)_2_	Propolis	Ca(OH)_2_
10^th^ day	25.00%	62.50%	37.50%	54.50%
15^th^ day	36.25%	54.50%	33.00%	36.00%
30^th^ day	16.75%	20.00%	30.00%	20.00%


Although fewer stem cells were found in the Propolis groups compared to the calcium hydroxide controls at each time point, there was no significant difference between the two groups except at day 10 at which time there was a dominance of stem cells in calcium hydroxide control group.

## Discussion

Knowledge of pulp physiology has increased considerably in recent years and led to a better understanding of pulp healing. Nonetheless the criteria agreed to characterize successful direct pulp capping vary among authors. The clinical criteria for successful pulp capping are that the tooth is free of symptoms, there is adequate reaction to sensitivity tests and the tooth has a normal radiographic appearance. As inflammation complicates the healing of pulp, a critical evaluation of the results of pulp capping can only be made histologically. Following injury to mature tooth pulp, progenitor cells recruit the repair processes and differentiate into second generation odontoblasts. Although in general practice direct pulp capping (DPC) is usually considered to be a temporary treatment, it has been suggested as a permanent treatment (3, 7).

Calcium hydroxide compounds are the gold standard for vital pulp therapy in human teeth. The procedure is carried out on pulps which are contaminated with bacteria and where there is a potential risk of bacterial leakage along the restoration margins (15). Evidence shows limited effectiveness of calcium hydroxide to eliminate bacteria from human root canals completely (16, 17). Propolis is a good antimicrobial and antifungal agent (18). It breaks down bacterial cell walls, cytoplasm and prevents bacterial cell division (19).

According to Silva et.al Propolis compared to other experimental materials was the least irritant one which can make it a valuable alternative in endodontics (20). Shaher et.al treated the fibroblasts of the pulp and periodontal ligament with Propolis and concluded that this material is not toxic (21). Scheler et.al showed regeneration of the pulp by applying Propolis on injured dental pulp, (22) while calcium hydroxide caused necrosis of the pulp chamber (7). During the present study, there was no sign of pulp necrosis with Propolis, while necrosis was observed in about 25% of the control calcium hydroxide group.

In present study specimens capped with Propolis showed no inflammation which could be related to the anti-inflammatory property of this material. Flavonoids and caffeic acid present in Propolis are known to play an important role in reducing the inflammatory response by inhibiting the lipoxygenase pathway of arachidonic acid. Flavonoids and caffeic acid also aid the immune system by promoting phagocytic activities and stimulating cellular immunity (23).

Ansorge has shown the ability of Propolis to stimulate the production of transforming growth factor (TGF) Beta 1 which is important for the differentiation of odontoblasts (24). It also induces the synthesis of collagen by dental pulp cells (25). However in this study there was no significant difference in the presence of odontoblasts between the two materials.

Bretz et.al reported formation of reparative dentin in DPC with Propolis (25). In another study Sabir et.al found partial dental bridge formation after 4 weeks with Propolis (26). Parolia et.al conducted a study on permanent teeth. They concluded that dentinal bridge formation and tubular dentin were more evident in Propolis and MTA (Mineral Trioxide Aggregates), whereas most of the calcium hydroxide specimens showed incomplete bridge formation with amorphous and non-tubular dense dentin (27). In other studies incomplete formation of dentinal bridge and tunnel defects have been reported in cases treated with calcium hydroxide (3, 7). Treatment with Propolis in the present study was associated with the formation of tubular dentin with no pores or connective tissue and which was similar to primary dentin (Fig 1). In contrast, the dentin formed by calcium hydroxide was porous, was filled with loose connective tissue and blood vessels and was similar to in structure to bone (Fig 2). Probably, this is due to the rapid cell turnover in guinea pig that results in the changing of SCs to mature cells after using bioactive Propolis on pulp during the experimental period.

Since dentin formed under two capping materials has different qualities, it seems that there are different odontoblast-like cells in each group, which may be due to the variable origins of their stem cells. This result is in agreement with Ji et al who observed that calcium hydroxide can stimulate DPSCs and periodontal ligaments (PDL) stem cells, producing hard tissue in exposed pulp tissue(3). Horsted-Bindslev et al proposed that perivascular cells and other cell populations, including bone marrow stem cells, which migrate via the blood stream may act as progenitor cells (7).

Stem cells can not be identified with certainty in any tissue: scientists rely on indirect properties such as the expression of repertoire surface proteins, clonogenicity or an undifferentiated state (1, 28). In this study presence of stem cells was evaluated using CD_29_ and CD_146_ markers which are specific for DPSCs (29).

Alliot showed that signals from calcium hydroxide can precipitate the differentiation of stem cells to odontoblasts (30). In the present study, stem cells were similarly detected using specific markers after capping by calcium hydroxide during the experimental period. To date there appears to have been no study published that has documented the stimulation of stem cells by Propolis. Thus, to our knowledge this is the first time that this finding has been reported.

This study showed that although more stem cells were found in the calcium hydroxide control group, compared with the Propolis group at each time point, the dentin which is formed by Propolis is of better quality. This may be due to the longevity of Propolis in situ compared with calcium hydroxide, which enables it to act as a stimulant for stem cell differentiation over a longer period.

Tecles et al. demonstrated that pulpal injury stimulated the proliferation of stem cells localized in the perivascular area (31). This result has been confirmed by other researchers showing that pericytes could also differentiate into odontoblasts (5, 29, 30 , 32). The present study showed that the number of perivascular stem cells at any given time was more than the number of dentin stem cells in both the Propolis and calcium hydroxide groups. Also there were some cases in which stem cells were completely absent even on the 10^th^ day. However, in these cases a dentinal barrier had already been formed. This may be explained by the known, rapid formation of dentin in guinea pigs.

## Conclusion

To our knowledge this is the first study that has documented the stimulation of stem cells by Propolis. It also provides evidence that Propolis has advantages over calcium hydroxide as a capping agent in vital pulp therapy. In addition to producing no pulpal inflammation, infection or necrosis, this material induces the production of a tubular and high quality dentin.
